# Influence of Sulfur Fumigation on Angelicae Dahuricae Radix: Insights from Chemical Profiles, MALDI-MSI and Anti-Inflammatory Activities

**DOI:** 10.3390/molecules30010022

**Published:** 2024-12-25

**Authors:** Changshun Wang, Yongli Liu, Xiaolei Wang, Zhenhe Chen, Zhenxia Zhao, Huizhu Sun, Jian Su, Ding Zhao

**Affiliations:** 1 College of Pharmacy, Hebei Medical University, Shijiazhuang 050017, China; shunzi20080508@126.com; 2 NMPA Key Laboratory for Quality Monitoring and Evaluation of Traditional Chinese Medicine (Chinese Materia Medica), Hebei Institute for Drug and Medical Device Control, Shijiazhuang 050227, China; liuyongli2008@126.com (Y.L.); 18603318068@163.com (X.W.); zhaozhenxia@hbyxjy.org.cn (Z.Z.); sunhuizhu@163.com (H.S.); sujian@hbyxjy.org.cn (J.S.); 3 Shimadzu China Innovation Center, Shimadzu China, Beijing 100020, China; spkczh@shimadzu.com.cn

**Keywords:** Angelicae Dahuricae Radix, sulfur fumigation, MALDI-MSI, in vitro anti-inflammatory effects, metabolites in blood, quality markers

## Abstract

Background: Angelicae Dahuricae Radix (ADR) is used as both a traditional Chinese medicine and a food ingredient in China and East Asian countries. ADR is generally sun-dried post-harvest but is sometimes sulfur-fumigated to prevent decay and rot. Although there are some studies on the effect of sulfur fumigation on ADR, they are not comprehensive. Methods: This study used HPLC fingerprinting, matrix-assisted laser desorption/ionization mass spectrometry imaging (MALDI-MSI), in vitro anti-inflammatory assays, and metabolite analysis in blood based on UPLC-MS/MS to assess the impact of sulfur fumigation on the active ingredients of ADR. Results: There were significant decreases in specific coumarins and amino acids, particularly byakangelicol, oxypeucedanin, L-proline, and L-arginine, following sulfur fumigation. Among the 185 metabolites in blood, there were 30 different compounds, and oxypeucedanin was the most obvious component to decrease after sulfur fumigation. ADR showed anti-inflammatory activity regardless of sulfur fumigation. However, the effects on the production of cytokines in LPS-induced RAW264.7 cells were different. Conclusions: Chemometric analysis and in vitro anti-inflammatory studies suggested that byakangelicol and oxypeucedanin could serve as potential quality markers for identifying sulfur-fumigated ADR. These findings provide a chemical basis for comprehensive safety and functional evaluations of sulfur-fumigated ADR, supporting further research in this field.

## 1. Introduction

Sulfur fumigation is widely applied to protect foods and herbal medicines from insects, fungi, and moisture during storage and transportation [1,2,3]. Despite its effectiveness in preservation, the safety risks associated with sulfur fumigation cannot be ignored. Sulfur fumigation can result in high levels of SO_2_ and heavy metal residues in herbal medicines, posing direct health risks. Recent studies show that sulfur fumigation alters the active ingredient content and compound structures in certain herbal medicines, which can affect their pharmacokinetics and pharmacological properties, potentially leading to severe toxicity [4,5,6]. Additionally, the SO_2_ residues in sulfur-fumigated herbal medicines gradually decrease over time, suggesting that quality evaluation should consider not only SO_2_ levels but also chemical composition changes. Identifying specific markers of sulfur fumigation is essential for developing a scientific evaluation method [7,8,9,10].

Angelicae Dahuricae Radix (ADR) is derived from the roots of *Angelica dahurica* (Fisch.ex Hoffm.) Benth. et Hook. f. or *Angelica dahurica* (Fisch. ex Hoffm.) Benth. et Hook. f. var. *formosana* (Boiss.) Shan et Yuan, both belonging to the Umbelliferae family [11]. It is recorded in the Shennong Materia Medica Classic as a mid-tier medicinal product and is a widely used traditional Chinese medicine with both medicinal and culinary applications [12]. ADR is known for alleviating colds, dispelling wind, relieving pain, promoting nasal clearance, addressing dryness and dampness, reducing swelling, and discharging pus. Plants of the Angelica genus have abundant pharmacological activities. As a plant of the Angelica genus [13], modern pharmacological studies indicate that ADR exhibits various biological activities, including analgesic [14], anti-inflammatory [15,16], antioxidant [17], anti-tumor [18], and whitening properties, contributing to its extensive use in medicine, food, cosmetics, and healthcare products [19].

ADR contains a high starch content, which can lead to rapid decay if not dried promptly. Therefore, sulfur fumigation is commonly used in the drying process of ADR. However, sulfur fumigation significantly affects the quality of ADR. Industrial sulfur is usually used in the fumigation process of medicinal materials, and industrial sulfur mostly contains Hg, Cd, As, and other heavy metal elements. During the combustion process, sulfur dioxide can be produced and toxic substances such as As and Hg are released. Therefore, although sulfur fumigation accelerates the drying and enhances the appearance of ADR, it has many hidden risks. Many studies report that it significantly reduces active ingredient levels, compromises pharmacological activity, and increases residues of sulfur dioxide, heavy metals, and harmful elements. This poses potential risks to the environment, respiratory and digestive tracts, liver, kidneys, and eyes [20]. Although some research has explored the effects of sulfur fumigation on ADR components [1,21], comprehensive investigation is still lacking.

Matrix-assisted laser desorption/ionization mass spectrometry imaging (MALDI-MSI) is a technique that desorbs and ionizes analytes from surfaces using laser irradiation, followed by mass spectrometry to determine the spatial distribution of target compounds [22]. Research has examined the spatial distribution of coumarins in the primary and lateral roots of ADR using MALDI-MSI [23]. However, no studies have investigated the microscopic imaging differences in ADR before and after sulfur fumigation.

In this study, an HPLC method was developed for the simultaneous quantification of ten coumarin components. Stoichiometric methods, including similarity analysis (SA), cluster analysis (CA), principal component analysis (PCA), and orthogonal partial least squares discriminant analysis (OPLS-DA), were subsequently used to comprehensively analyze the identified furanocoumarin components. Subsequently, MALDI-MSI was employed to elucidate the spatial distribution of changes in coumarin metabolites before and after sulfur fumigation. Finally, the in vitro anti-inflammatory activity of differential compounds before and after sulfur fumigation was investigated. Specific markers for identifying sulfur-fumigated ADR were established, providing a scientific basis for more comprehensive and systematic evaluations of ADR quality.

## 2. Results and Discussion

### 2.1. HPLC Fingerprint of Sulfur-Fumigated and Non-Sulfur-Fumigated ADR

**Figure 1:**
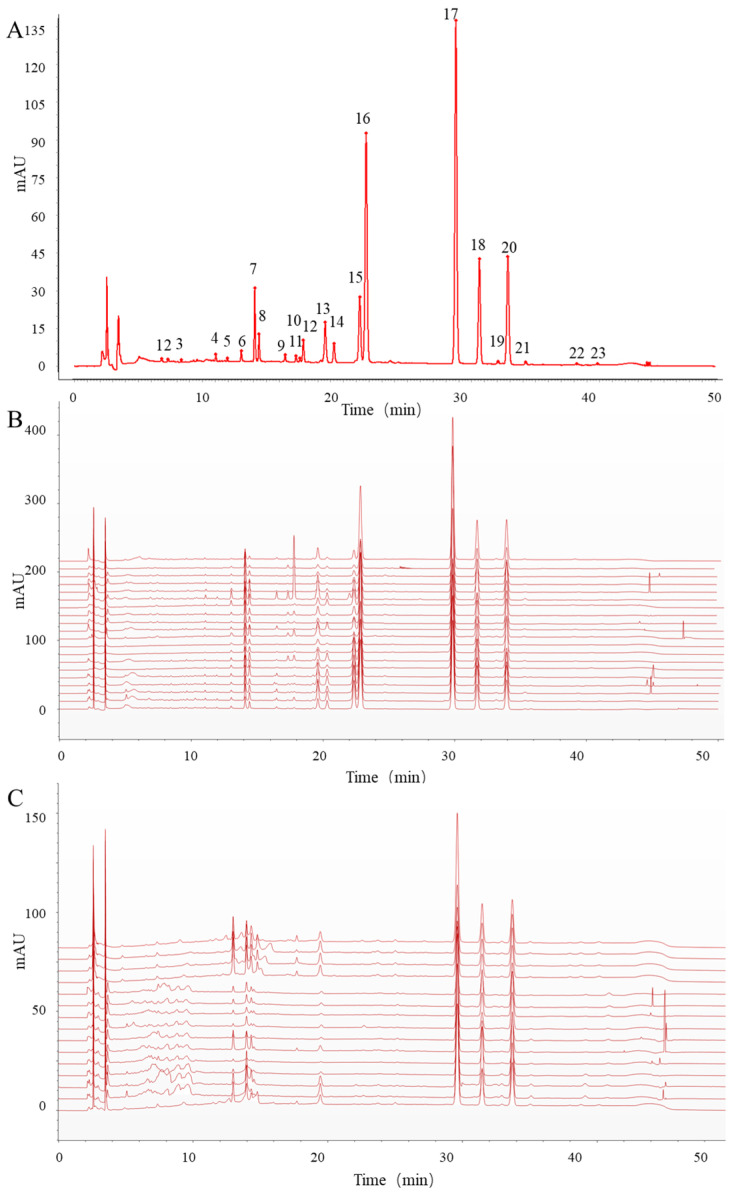
The fingerprints of reference compounds (**A**), non-fumigated ADR (20 batches) (**B**), and fumigated ADR (15 batches) (**C**).

Fingerprint technology is essential for quality control in traditional Chinese medicine [24]. The fingerprints of both non-sulfur-fumigated and sulfur-fumigated ADR are illustrated in Figure 1. The reference fingerprint is depicted in Figure 1A, while the HPLC chromatogram of mixed reference substances is shown in Figure S1. The fingerprint identified a total of 23 common peaks, which correspond to ten calibrated components. The chromatographic conditions resulted in a rapid analysis time, symmetric and narrow peak shapes, and a separation factor greater than 1.6 for reference compounds.

Similarity analysis of fumigated and non-fumigated ADR was performed using the ‘Similarity Evaluation System of Traditional Chinese Medicine Chromatographic Fingerprint’ (version 2008). First, the chromatograms of 20 batches of non-fumigated ADR were imported into the software in AIA (*.cdf) format to establish the reference chromatogram. Subsequently, all fumigated and non-fumigated ADR samples were compared with the reference chromatogram, and similarity values were calculated as shown in Table S1. A significant difference in the number of chromatographic peaks was observed between fumigated and non-fumigated samples (*p* < 0.01).

**Figure 2:**
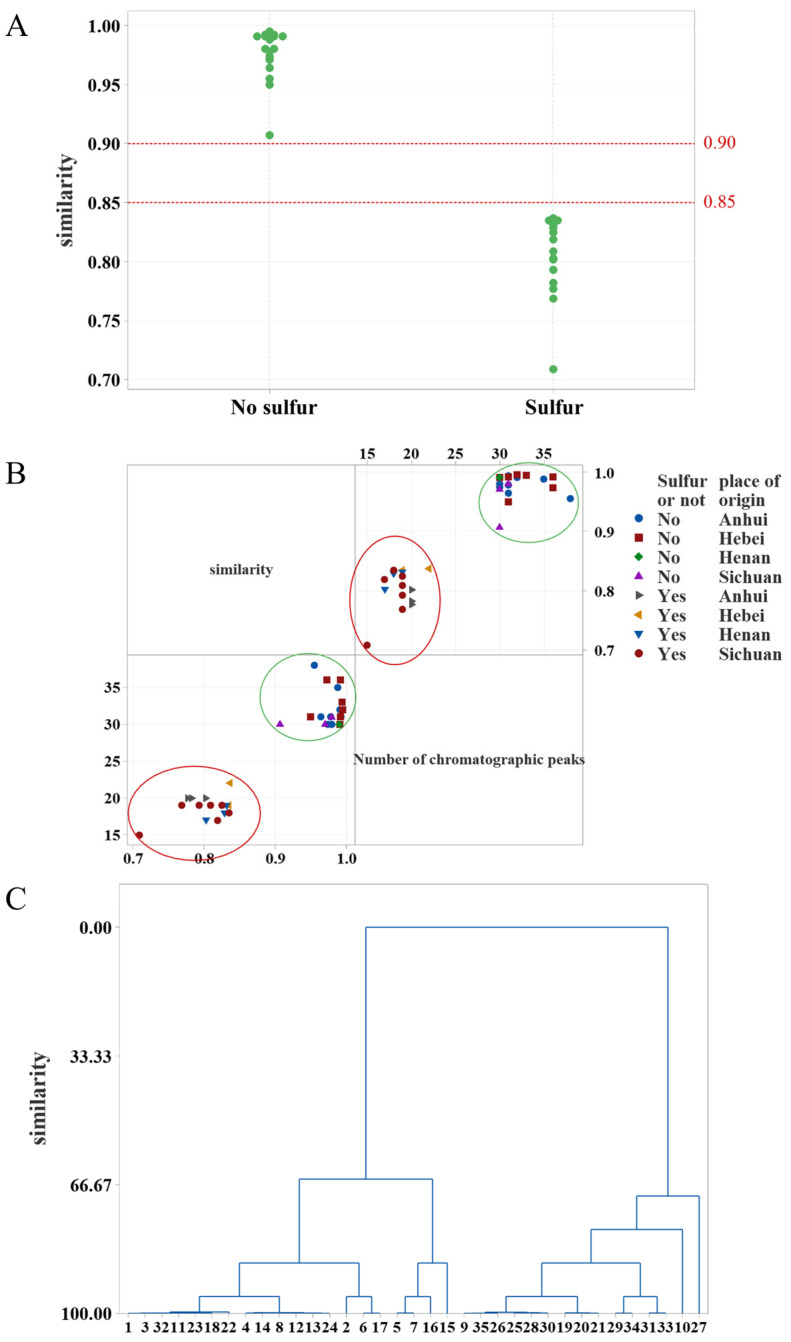
Similarity analysis diagram of ADR. (**A**) Individual Value Plot of similarity of ADR. (**B**) Matrix graph of similarity and chromatographic peak number. (**C**) HCA of ADR.

Non-fumigated ADR exhibits more than 30 peaks and a similarity value ranging from 0.907 to 0.995. In contrast, fumigated ADR has fewer than 22 peaks and a similarity value ranging from 0.709 to 0.837 (Figure 2A). The similarity of ADR without sulfur fumigation was higher than 0.90, and after sulfur fumigation, the similarity decreased to less than 0.85. The matrix diagram revealed that fumigated and non-fumigated ADR formed two distinct clusters based on both chromatographic peak numbers and similarity values (Figure 2B). ADR without sulfur fumigation exhibited a chromatographic peak number above 30; after sulfur fumigation, certain chromatographic peaks were missing, resulting in a number of chromatographic peaks at 22 or below. Similarity values were analyzed using MINITAB 21 for cluster analysis. The clustering diagram was generated using the inter-group linkage method and squared Euclidean distance as the measurement metric (Figure 2C). The 35 batches of ADR were automatically clustered into two groups. Batches S9–S10, S19–S21, S25–S31, and S33–S35 were grouped as non-fumigated ADR, while the remaining batches were classified as fumigated ADR, consistent with the similarity matrix diagram.

Sulfur fumigation is commonly employed to dry ADR and can significantly impact its quality. While many studies have reported on ADR fingerprints [25], there is limited research on sulfur-fumigated ADR. Numerous chromatographic peaks were clearly absent in ADR following sulfur fumigation (Figure 1C).

### 2.2. Difference in Active Ingredients in Sulfur-Fumigated and Non-Sulfur-Fumigated ADR

**Figure 3:**
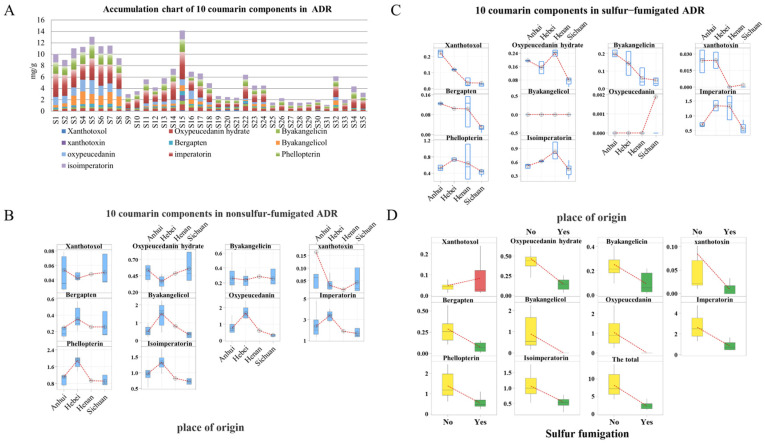
Content of coumarins in ADR. (**A**) Accumulation chart of 10 coumarin components in ADR; (**B**) box plot of 10 coumarins in non-sulfur-fumigated ADR; (**C**) box plot of 10 coumarins in sulfur-fumigated ADR; (**D**) effects of sulfur fumigation on coumarins in ADR.

The contents of 10 coumarins were compared for the 35 batches of ADR (Table S2 and Figure 3). From the accumulation chart (Figure 3A), it can be seen that there were differences in the content of ADR in different batches. The average contents of 10 coumarins in ADR showed regional differences (Figure 3B,C). The red lines represent the mean values of the components, They pointed out that the levels of bergapten, byakangelicol, oxypeucedanin, imperatorin, phellopterin, and isoimperatorin exhibited a declining trend in Hebei, Anhui, Henan, and Sichuan (Figure 3B). Furthermore, the levels of xanthotoxol, byakangelicin, and xanthotoxin in ADR were relatively low. Many studies have shown that sulfur fumigation notably impacts the quality of ADR, especially coumarin content, with the exception of xanthotoxol [26]. The box plot in Figure 3D demonstrates the effect of sulfur fumigation on the coumarin constituents of ADR. As seen in the figure, the content of xanthotoxol (marked in red in Figure 3D) increased after sulfur fumigation, while the content of other components decreased significantly. Byakangelicol and oxypeucedanin almost disappeared in ADR after sulfur fumigation, suggesting that sulfur fumigation primarily affected these two components.

### 2.3. Principal Component Analysis (PCA) and Orthogonal Component Analysis Orthogonal Partial Least Squares-Discriminant Analysis (OPLS-DA)

**Figure 4:**
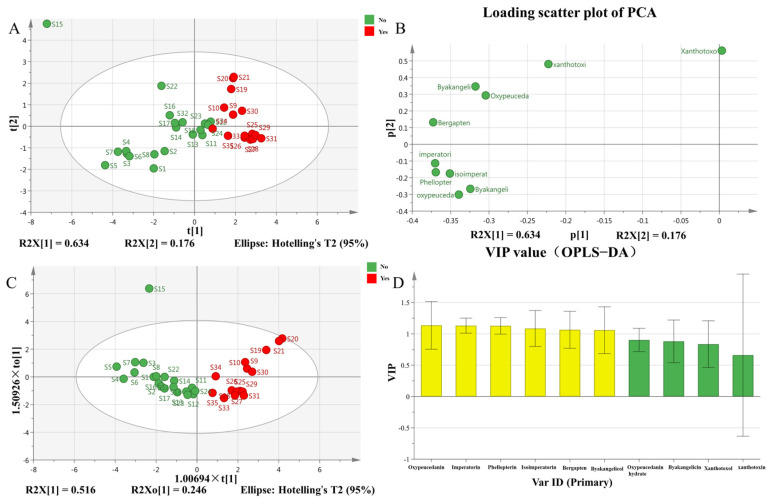
PCA and OPLS-DA analysis of ADR. (**A**) Score scatter plot of PCA; (**B**) loading scatter plot of PCA; (**C**) score scatter plot of OPLS-DA; (**D**) VIP value.

PCA was performed on the contents of 10 coumarin components from 35 batches of ADR collected from different habitats. As an unsupervised learning method, PCA effectively grouped fumigated and non-fumigated ADR. The first and second principal components accounted for a cumulative variance contribution rate of 81.0%, indicating that these components effectively reflect the differences between fumigated and non-fumigated ADR. The PCA score plot (Figure 4A) shows distinct clustering of sulfur-fumigated and non-sulfur-fumigated ADR, consistent with the HCA findings. In the loading plot (Figure 4B), bergapten, byakangelicol, oxypeucedanin, imperatorin, phellopterin, and isoimperatorin exhibited larger absolute values in the coordinate system, indicating their significant contribution to the overall quality of ADR.

OPLS-DA analysis was performed using Simca 14.1 software (Figure 4C) to identify components that significantly influence the quality of various ADR batches. We further screened chromatographic peaks with Variable Importance in Projection (VIP) values greater than 1.0 (Figure 4D). Six significant variables were identified: oxypeucedanin, imperatorin, phellopterin, isoimperatorin, bergapten, and byakangelicol. These findings indicate that these six compounds are key markers of ADR. Therefore, it is important to monitor the quality changes in these compounds during the quality control and evaluation of ADR.

**Figure 5:**
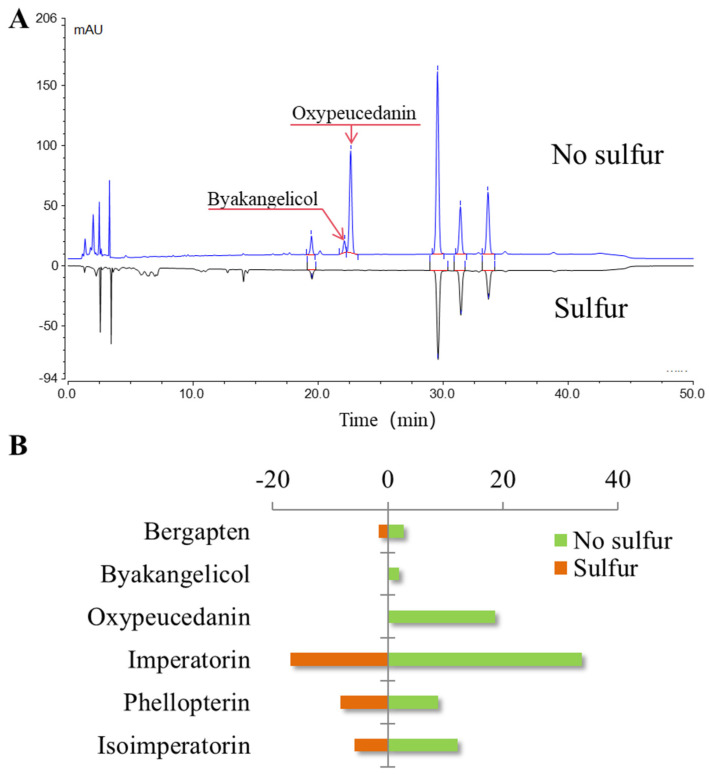
Comparison between natural drying and sulfur fumigation of ADR. (**A**) Comparison of chromatographic peaks between naturally dried and sulfur-fumigated ADR; (**B**) comparison of chromatographic peak areas of six markers between sulfur-fumigated and non-sulfur-fumigated ADR.

ADR was cultivated in a well-known production area (Anguo, China). Subsequently, both natural drying and sulfur fumigation were applied to ADR cultivated in the same area. Figure 5 compares HPLC chromatographic peaks and the peak areas of the six markers. As shown in the figure, sulfur fumigation resulted in the loss of byakangelicol and oxypeucedanin. This phenomenon may result from hydrolysis reactions of coumarins and other lactone-containing components in ADR under acidic conditions. These components may serve as markers to indicate whether ADR has undergone excessive sulfur fumigation.

### 2.4. MALDI-MSI Analysis of Active Ingredients in ADR

**Figure 6:**
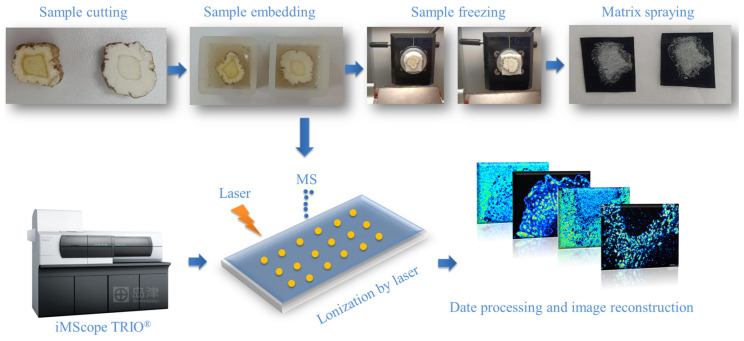
Experimental procedure of mass spectrometry imaging for ADR.

The spatial distribution of active ingredients was investigated, and Figure 6 illustrates the general analysis procedure using MALDI-MSI. The average mass spectrum for fumigated and non-fumigated ADR in positive ion mode is shown in Figure S4. Positive ions were predominantly detected in the mass ranges of *m*/*z* 100–400 and *m*/*z* 400–800. The tentative identification of components was based on accurate mass-to-charge ratios, referencing isotopic peaks, reference standards, the literature, and databases. Table S3 summarizes the compounds identified in ADR, including coumarins, flavonoids, phenylpropanoids, and amino acids.

**Figure 7:**
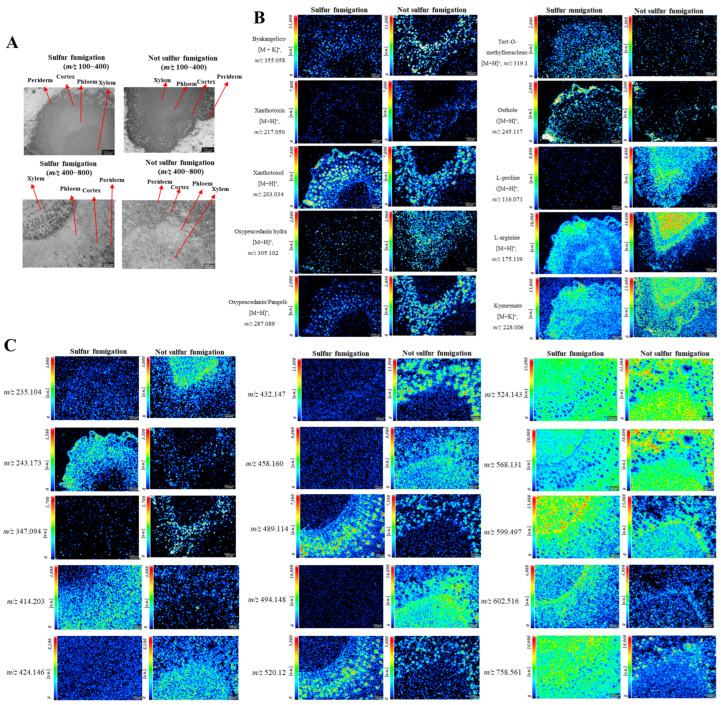
Spatial distribution of typical ingredients in fumigated and non-fumigated ADR. (**A**) Optical image of ADR slices; (**B**) distribution of ingredients identified; (**C**) distribution of some unknown components.

Optical images of fumigated and non-fumigated ADR slices are shown, where the substructures (periderm, cortex, phloem, and xylem) can be identified. The distinct spatial distribution of various characteristic constituents is also illustrated in Figure 7B (identified) and Figure 7C (unknown). Coumarins are the most abundant class of chemical components in ADR. They were primarily detected as positive ions and displayed diverse distributions. As shown in Figure 7B, furanocoumarins such as byakangelicol ([M+K]^+^, *m*/*z* 355.058), xanthotoxin ([M+H]^+^, *m*/*z* 217.050), xanthotoxol ([M+H]^+^, *m*/*z* 203.034), and oxypeucedanin ([M+H]^+^, *m*/*z* 287.089) were exclusively located in the periderm, cortex, and phloem, with the highest content found in the phloem and almost none in the xylem. Tert-O-methylheraclenol ([M+H]^+^, *m*/*z* 287.089) is mainly distributed in the cortex, phloem, and xylem regions. Osthole ([M+H]^+^, *m*/*z* 245.117) is primarily found in the periderm and cortex. Clearly, most furanocoumarins showed the highest abundance in the phloem and cortex. Other compounds, including *L*-proline ([M+H]^+^, *m*/*z* 116.071), *L*-arginine ([M+H]^+^, *m*/*z* 175.119), and kynurenate ([M+K]^+^, *m*/*z* 228.006), are primarily distributed throughout the cross-sectional regions.

Figure 7 shows the signal strength of identified compounds in ADR before and after sulfur fumigation using MALDI-MSI. After sulfur fumigation, there was a significant decrease in coumarins such as byakangelicol, xanthotoxin, oxypeucedanin hydrate, and oxypeucedanin/pangel. In contrast, melatonin, xanthotoxol, tert-o-methylheraclenol, and osthole exhibited an increase. Additionally, other chemicals in ADR, including *L*-proline, kynurenate, and *L*-arginine, experienced significant decreases. Furthermore, some unknown compounds, including *m*/*z* 235.104, *m*/*z* 347.094, *m*/*z* 424.146, *m*/*z* 432.147, *m*/*z* 458.160, and *m*/*z* 494.148, decreased significantly after sulfur fumigation. Meanwhile, other compounds increased significantly after sulfur fumigation, including *m*/*z* 243.173, *m*/*z* 414.203, *m*/*z* 489.114, *m*/*z* 520.127, *m*/*z* 602.516, and *m*/*z* 758.561.

Sulfur fumigation also influenced the distribution of components in ADR. Significant changes were noted for some known components after sulfur fumigation. Regarding the pharmaceutical active ingredients, xanthotoxol increased and was more prominent in the xylem, while oxypeucedanin was lost in both the periderm and phloem; osthole was found throughout the slice but only in the periderm without fumigation. Tert-*O*-methylheraclenol also increased in the periderm, cortex, phloem, and xylem but was significantly lower without fumigation. The amino acids, such as *L*-proline, *L*-arginine, and kynurenine, were distributed in the whole slice without fumigation, and *L*-arginine was observed higher in the xylem than other parts. After fumigation, *L*-proline almost disappeared, while both *L*-arginine and kynurenine shifted to the periderm and cortex and were significantly less present in the xylem. An uneven distribution was also noted for some unidentified components after sulfur fumigation. For instance, *m*/*z* 235.104 and *m*/*z* 424.146 were evenly distributed and decreased significantly throughout the slice but were more prominent in the xylem without fumigation. *m*/*z* 432.147 almost disappeared but was observed in the periderm, cortex, and phloem without fumigation. *m*/*z* 243.173 is distributed in the periderm, cortex, and phloem with significantly increased content but is present at lower levels in the cortex and phloem without fumigation. *m*/*z* 494.148 disappeared but was distributed in the phloem and xylem without fumigation. *m*/*z* 599.497 and *m*/*z* 758.561 increased sharply, with the highest content found in the xylem and distributed throughout the slice. In summary, sulfur fumigation significantly alters the distribution of components in ADR, which can be intuitively observed using MALDI-MSI, and similar changes were noted with fingerprint analysis.

**Figure 8:**
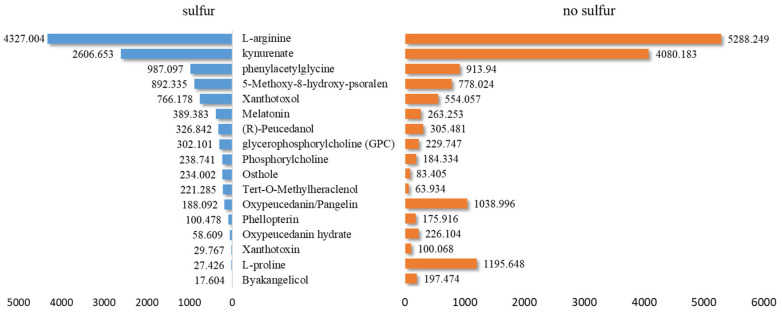
Signal strength of identified compounds in ADR before and after sulfur fumigation by MALDI-MSI.

The mass spectrum response values for some analyzed components are presented in Table S4. According to the data, a bar chart was created (Figure 8). Paired *t*-test analysis indicated a significant difference in the MALDI-MSI response values before and after sulfur fumigation (*p* < 0.001).

### 2.5. Byakangelicol, Oxypeucedanin, and ADR Prevent LPS-Induced Inflammation In Vitro

**Figure 9:**
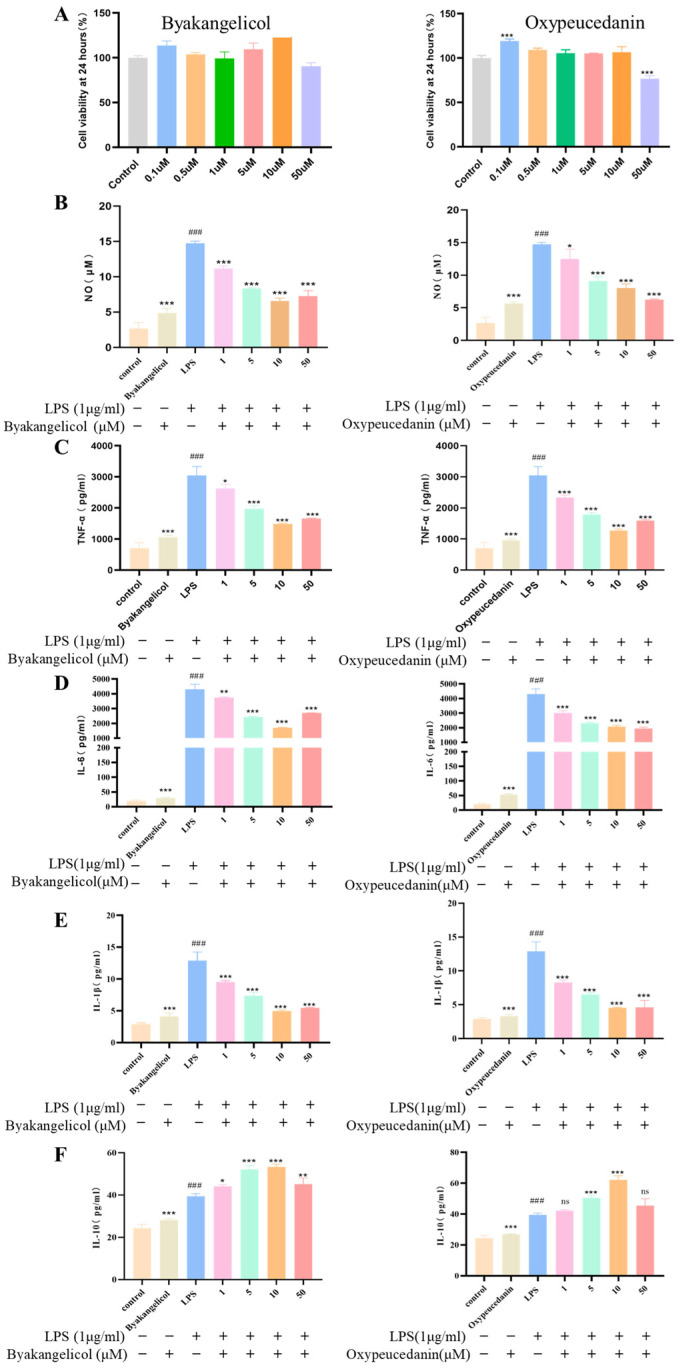
In vitro validation of byakangelicol and oxypeucedanin. (**A**) Effects of byakangelicol and oxypeucedanin on the viability of RAW264.7 cells; effect of byakangelicol and oxypeucedanin on the production of NO (**B**), TNF-α (**C**), IL-6 (**D**), IL-1β (**E**), and IL-10 (**F**) in LPS-induced RAW264.7 cells. The results represent the mean ± SEM (n = 3), * *p* < 0.05, ** *p* < 0.01, *** *p* < 0.001 (vs. LPS), ### *p* < 0.001 (vs. Control), ns: not significant.

Chemometric analysis indicates that sulfur fumigation is the primary factor influencing the quality of ADR. Byakangelicol and oxypeucedanin were absent in sulfur-fumigated samples, indicating their potential as markers for sulfur fumigation in ADR. The viability of RAW264.7 cells was assessed using a CCK-8 assay to evaluate the effects of byakangelicol and oxypeucedanin. Figure 9A shows that byakangelicol and oxypeucedanin exhibited no cytotoxic effects on RAW264.7 cells at concentrations ranging from 0.1 to 50 μM, except for a slight decrease in cell activity at 50 μM. Figure 9B indicates that the level of NO in the cell supernatant of the model group significantly increased after LPS stimulation. Compared to the model group, byakangelicol and oxypeucedanin significantly reduced the concentrations of NO in a dose-dependent manner.

Inflammation is a pathological process in various diseases and represents the most primitive protective response to a range of stimuli. Macrophages are key players in the innate and adaptive immune responses [27], capable of transforming between M1 and M2 phenotypes in specific microenvironments. LPS can polarize macrophages to the M1 phenotype, which is responsible for pro-inflammatory responses and the release of pro-inflammatory mediators and cytokines such as TNF-α, IL-6, and IL-1β. In contrast, M2 macrophages promote tissue repair by inhibiting the inflammatory response through the release of mediators such as IL-10 [28,29]. Byakangelicol and oxypeucedanin significantly reduced the concentrations of pro-inflammatory cytokines (TNF-α, IL-6, and IL-1β) in each treatment group compared to the LPS group, while the content of the anti-inflammatory cytokine IL-10 significantly increased (Figure 9C–F). It is concluded that byakangelicol and oxypeucedanin exhibit strong anti-inflammatory activity, potentially promoting inflammatory repair by down-regulating the levels of pro-inflammatory cytokines TNF-α, IL-6, and IL-1β, while up-regulating the level of the anti-inflammatory cytokine IL-10.

**Figure 10:**
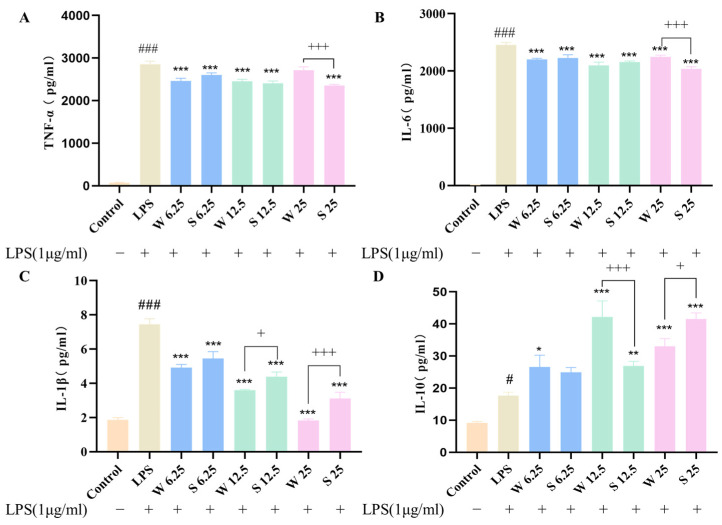
In vitro validation of ADR before and after sulfur fumigation. Effect of ADR before and after sulfur fumigation on the production of TNF-α (**A**), IL-6 (**B**), IL-1β (**C**), and IL-10 (**D**) in LPS-induced RAW264.7 cells. The results represent the mean ± SEM (n = 3), * *p* < 0.05, ** *p* < 0.01, *** *p* < 0.001 (vs. LPS), ^#^ *p* < 0.05, ^###^ *p* < 0.001 (vs. Control), ^+^ *p* < 0.05, ^+++^ *p* < 0.001 (non-sulfur-fumigated ADR vs. sulfur-fumigated ADR).

There are rich coumarins in ADR. Coumarins and isocoumarins are known to act as anti-inflammatory molecules [19,30]. Moreover, the anti-inflammatory activity of ADR before and after sulfur fumigation was also investigated. ADR showed anti-inflammatory activity regardless of sulfur fumigation. However, the effects of non-sulfur-fumigated ADR and sulfur-fumigated ADR on the production of cytokines TNF-α, IL-6, IL-1β, and IL-10 in LPS-induced RAW264.7 cells were different (Figure 10). Non-sulfur-fumigated ADR showed a better reduction effect than sulfur-fumigated ADR for TNF-α at 6.25 μg/mL (Figure 10A) and for IL-6 at 6.25 μg/mL, 12.5 μg/mL (Figure 10B). For the cytokine IL-1β, non-sulfur-fumigated ADR (W 6.25, W 12.5, W 25) showed better effects on reducing inflammatory factors (Figure 10C). Non-sulfur-fumigated ADR showed a better elevation of anti-inflammatory cytokine IL-10 with the concentration of 6.25,12.5 μg/mL (Figure 10D). However, high concentration of sulfur-fumigated ADR (S 25 μg/mL) also showed good effects of down-regulating the levels of pro-inflammatory cytokines TNF-α and IL-6, while up-regulating the level of the anti-inflammatory cytokine IL-10. This indicated that although sulfur fumigation leads to the reduction in or disappearance of some components in ADR, the high concentration of sulfur-fumigated ADR still had some anti-inflammatory activity, but it cannot be shown that sulfur fumigation is reliable, and a comprehensive investigation is still needed.

### 2.6. Difference in Metabolites in Blood in Sulfur-Fumigated and Non-Sulfur-Fumigated ADR

**Figure 11:**
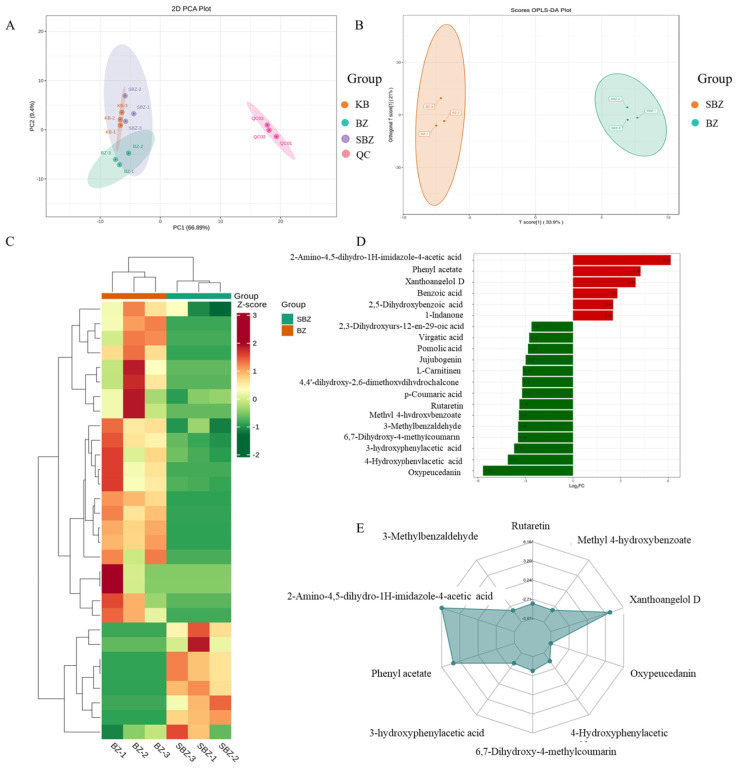
Difference in metabolites in blood in sulfur-fumigated and non-sulfur-fumigated ADR. (**A**) PCA; (**B**) OPLS-DA analysis; (**C**) heatmap cluster of ADR; (**D**) fold change bar chart; (**E**) radar chart.

Due to the complex composition of traditional Chinese medicine (TCM), it is difficult to elucidate the active ingredients that exert the therapeutic effect. Therefore, it is important to determine the material basis and in vivo process of TCM. Because of the influence of sulfur fumigation on the quality of ADR, it is necessary to study the differences in its components and metabolites in blood before and after sulfur fumigation. Through UPLC-MS/MS analysis, 185 metabolites were detected, including coumarins, terpenes, alkaloids, flavonoids, phenolic acids, steroids, etc. Among them, the coumarin components included 4-Hydroxycoumarin, 6,7-Dihydroxy-4-methylcoumarin, 7,8-Dihydroxy-4-phenylcoumarin, Decursinol, Heraclenol, Oxypeucedanin, Oxypeucedanin hydrate, Rutaretin, etc. It should be noted that Oxypeucedanin and Oxypeucedanin hydrate were the prototype components of ADR. The metabolites were analyzed using chemometrics. The results of PCA showed the trend of metabolome separation among the groups, indicating that there were differences between group BZ and group SBZ (Figure 11A). That was further confirmed by OPLS-DA analysis and heatmap cluster (Figure 11B,C). Differential metabolites of ADR before and after sulfur fumigation were screened, and there were 30 different compounds in blood metabolism, including 9 phenolic acids, 2 flavonoids, 6 coumarins, 5 alkaloids, 4 triterpenoids, 1 ketone, and 3 aldehydes. Among the differential metabolites, 8 were up-regulated after sulfur fumigation and 22 were down-regulated (Table S5). Among the differential metabolites, the top 20 metabolites with the largest absolute value of log_2_FC were selected for fold change bar chart (Figure 11D), and 16 metabolites were down-regulated. The top ten metabolites with the largest absolute value of log_2_FC were selected for radar map drawing (Figure 11E), and seven metabolites were down-regulated after sulfur fumigation. The component with the greatest change was oxypeucedanin. It was reduced by about 50 times after sulfur fumigation. The result was similar to that of fingerprinting. It is speculated that the chemical composition of ADR was destroyed after sulfur fumigation, and the composition content is reduced, and the composition content is relatively low after entering the blood.

## 3. Materials and Methods

### 3.1. Plant Materials

Thirty-five batches of ADR were obtained from medicinal material markets in Hehuachi, Bozhou, Anguo, and Yuzhou, China (Table 1). All samples were collected during the autumn harvest period and purchased in the market after processing at the origin. After purchase, they were stored in our laboratory and kept insect-proof and moisture-proof. One batch of ADR was cultivated in Anguo, with half naturally dried and the other half sulfur-fumigated after harvest. The sulfur dioxide residue of sulfur-fumigated ADR was 378 mg/kg. All samples were authenticated as *Angelica dahurica* (Fisch. ex Hoffm.) Benth. et Hook. f. var. *formosana* (Boiss.) Shan et Yuan by Prof. Ding Zhao from the department of pharmacognosy, Hebei Medical University. The specimens were stored at the NMPA Key Laboratory for Quality Monitoring and Evaluation of Traditional Chinese Medicine, Hebei Institute for Drug and Medical Device Control.

### 3.2. Chemicals and Reagents

Standards with a purity above 98% were sourced from different suppliers. Imperatorin (Lot No. 110826-201918) and isoimperatorin (Lot No. 110827-202113) were purchased from the National Institutes for Food and Drug Control (Beijing, China). Xanthotoxol (Lot No. 3537), byakangelicin (Lot No. 8435), phellopterin (Lot No. 9345), byakangelicol (Lot No. 8966), and oxypeucedanin hydrate (Lot No. 10073), each with a purity above 98%, were obtained from Shanghai Standard Technology Co., Ltd., Shanghai, China. Bergapten (Lot No. DST210409-012) and xanthotoxin (Lot No. DST210407-062), both with purities over 98%, were acquired from Chengdu Desite Biotechnology Co., Ltd., Chengdu, China. Oxypeucedanin (Lot No. G22050128) with a purity above 98% was obtained from Tan Mo Quality Control Technology Co., Ltd., Changzhou, China. α-Cyano-4-hydroxycinnamic acid (CHCA, Lot No. MKBQ8235V) was purchased from Sigma-Aldrich, Darmstadt, Germany. HPLC-grade acetonitrile (ACN) and methanol (MeOH) were obtained from Fisher Scientific, Fair Lawn, NJ, USA, and analytical-grade methanol was obtained from Sinopharm Chemical Reagent Co., Ltd., Shanghai, China. Ultra-pure water was obtained from Watsons, Guangzhou, China. DMEM cell culture medium and LPS were obtained from Sigma-Aldrich. The CCK-8 Kit was purchased from Beijing Solarbio Science & Technology Co., Ltd., Beijing, China. TNF-α, IL-6, IL-10, and IL-1β ELISA kits were purchased from ABclonal Technology Co., Ltd., Wuhan, China. The Nitric oxide (NO) detection kit was obtained from Beyotime Biotech Inc., Shanghai, China.

### 3.3. Preparation of Sample Solutions and Standard Solutions

ADR was first ground into powder prior to extraction. Then, 0.2 g of the powder was placed into a 100 mL conical flask with a cover and sonicated with 25 mL of methanol for 60 min. After cooling to room temperature, the weight loss was compensated by adding methanol to the original volume. The sample solution was then filtered through a 0.22 μm membrane before analysis. The sample from Anhui (S16) was used for methodological validation.

Simultaneously, each standard, including xanthotoxol, oxypeucedanin hydrate, byakangelicin, xanthotoxin, bergapten, byakangelicol, oxypeucedanin, imperatorin, phellopterin, and isoimperatorin, was precisely weighed and dissolved in methanol to prepare individual stock solutions. These stock solutions were then diluted with methanol to prepare the mixed standard solution.

### 3.4. Chromatographic Conditions for Fingerprint Analysis and Quantitative Analysis

An Ultimate 3000 HPLC system (Thermo Fisher Scientific, Waltham, MA, USA), consisting of an autosampler, quaternary pump, column compartment, and diode array detector, was used for both fingerprint and quantitative analyses. Chromatographic separation was performed on a Waters Symmetry C18 column (250 mm × 4.6 mm, 5 μm, Waters Corp., Milford, MA, USA). The mobile phase consisted of acetonitrile (A) and water (B). The gradient elution program was as follows: 0–15 min, 10–47% A; 15–20 min, 47% A; 20–30 min, 47–60% A; 30–40 min, 60% A. Detection was carried out at a wavelength of 254 nm. The flow rate was maintained at 1.0 mL/min, with a sample injection volume of 10 µL.

Repeatability was assessed by analyzing nine samples at three concentration levels (50%, 100%, and 150%), each prepared and analyzed independently in triplicate. Accuracy was evaluated at three levels: high, moderate, and low. Additionally, the same solution was injected and measured at 0, 1, 2, 5, 12, 15, and 24 h to assess stability. Relative standard deviation (RSD) values were calculated for repeatability and stability assessments, yielding satisfactory results. Different instruments, columns, column temperatures, and flow rates were tested, demonstrating excellent applicability (Table S6).

### 3.5. Sample Preparation and MALDI-MSI Analysis

A slightly modified standard procedure was used to prepare ADR sections. Sulfur-fumigated and non-sulfur-fumigated ADR samples were cleaned and cut into 1 cm segments. The segments were embedded in 10% gelatin and stored at −80 °C for two hours. Sectioning was performed using a microtome (Leica CM1950, Nussloch, Germany). The sample was first attached to the holder with optimal cutting temperature compound (OCT), then placed on an ultralow-temperature quick-freezing table (−22 °C) in the slicer for 5 min prior to sectioning. Subsequently, 30 μm sections were prepared and transferred onto conductive glass slides. After vacuum drying for 20 min, the glass slide was fixed to the holder and transferred to an automatic matrix sublimation instrument (iMLayer, Shimadzu, Kyoto, Japan) for matrix application. α-Cyano-4-hydroxycinnamic acid (CHCA) was deposited at 250 °C with a thickness of 0.7 μm.

MALDI-MSI data were obtained using an imaging mass spectrometer (iMScope TRIO, Shimadzu, Kyoto, Japan) equipped with an ion trap/time-of-flight mass analyzer, integrated optical microscope, and a laser source (Nd at 355 nm). The detector voltage was set to 2.10 kV. Mass spectra were acquired from *m*/*z* 100 to 800 in positive ion mode using imaging MS Solution (ver. 1.30, Shimadzu). The laser diameter was set to 3 (40 μm in arbitrary units of the iMScope TRIO), with a repetition frequency of 1000 Hz, 100 shots/pixel, and an intensity setting of 70.

### 3.6. Cell Culture Assays

RAW264.7, a murine macrophage-like leukemia cell line, was obtained from Wuhan Pricella Biotechnology Co., Ltd. (Wuhan, China). Cells were cultured in basal DMEM supplemented with 10% heat-inactivated FBS, 100 μg/mL streptomycin, and 100 U/mL penicillin at 37 °C in a humidified incubator with 5% CO_2_. RAW264.7 cells were stimulated with 1 µg/mL LPS for 24 h to establish an in vitro inflammation model. A CCK-8 assay was conducted to evaluate the effects of byakangelicol and oxypeucedanin on cell viability. NO production was measured using the Griess method. IL-6, TNF-α, IL-1β, and IL-10 levels were quantified using ELISA.

### 3.7. Metabolite Analysis in the Blood

In total, 50 g of both sulfur-fumigated and non-sulfur-fumigated ADR samples were extracted twice using 70% methanol for 2 h each. The extracts were concentrated under reduced pressure and freeze-dried. The non-sulfur-fumigated ADR yielded 39.6% (*w*/*w*), while the sulfur-fumigated yielded 19.6% (*w*/*w*). The animal experiments were approved by Laboratory Animal Ethical and Welfare Committee of Hebei Medical University (Approval Code: IACUC-Hebmu-2023041). Nine SD rats were divided into three groups: blank (KB), non-sulfur-fumigated (BZ), and sulfur-fumigated (SBZ), with three rats per group. Rats received a 2 g/kg drug dose, and 0.5 mL blood samples were collected at various intervals up to 24 h post-administration. Blood was mixed with heparin, centrifuged, and the supernatant was frozen at −80 °C. Samples were later thawed, vortexed briefly, and 50 μL was mixed with 300 μL of 70% methanol in a centrifuge tube, then vortexed for 3 min, centrifuge for 10 min at 4 °C. Transfer 200 μL of supernatant to a 1.5 mL centrifuge tube and refrigerate at −20 °C for 30 min. Centrifuge at 12,000 r/min and 4 °C for 3 min. Remove 180 μL of supernatant for UPLC-MS/MS analysis. UPLC conditions: Agilent SB-C18 column (1.8 µm, 2.1 mm × 100 mm) with a mobile phase of pure water with 0.1% formic acid (A) and acetonitrile with 0.1% formic acid (B). Sample measurements were performed with a gradient program that employed the starting conditions of 95% A, 5% B. Within 9 min, a linear gradient to 5% A, 95% B was programmed, and a composition of 5% A, 95% B was kept for 1 min. Subsequently, a composition of 95% A, 5.0% B was adjusted within 1.1 min and kept for 2.9 min. The flow velocity was set as 0.35 mL per minute. The column oven was set to 40 °C, with the injection volume of 2 μL. The effluent was alternatively connected to an ESI-triple quadrupole-linear ion trap (QTRAP)-MS. The ESI source operation parameters were as follows: source temperature 550 °C; ion spray voltage (IS) 5500 V (positive ion mode)/−4500 V (negative ion mode); ion source gas I (GSI), gas II(GSII), and curtain gas (CUR) were set at 50, 60, and 25 psi, respectively; the collision-activated dissociation (CAD) was high. QQQ scans were acquired as MRM experiments with collision gas (nitrogen) set to medium.

### 3.8. Data Analysis

IMAGEREVEAL MS software (version 1.20, Shimadzu, Kyoto, Japan) was used for processing and visualizing MALDI-MSI data. During image registration, the optical image was aligned with the corresponding mass spectrometry image of the sample. Regions of interest (ROI) were defined based on the optical images. HPLC data were analyzed using Minitab 21 (Minitab Corp., State College, PA, USA) and SIMCA 14.1 (Umetrics Corp., SWE, Umea, Sweden). All experiments were independently repeated at least three times, and representative results are shown. Data are expressed as mean ± SD, and group comparisons were conducted using one-way ANOVA. Statistical significance was set at *p* < 0.05, *p* < 0.01, and *p* < 0.001. All statistical analyses were performed using GraphPad Prism (version 8.4.3.686, GraphPad Software Inc., La Jolla, CA, USA).

## 4. Conclusions

This study identified significant differences in the chemical constituents of ADR before and after sulfur fumigation, as analyzed by MALDI-MSI. Most coumarins and specific amino acids, including *L*-proline, oxypeucedanin hydrate, kynurenate, and *L*-arginine, showed a reduction in concentration. This reduction may be attributed to hydrolysis and addition reactions that occur under acidic conditions, causing coumarins and other compounds with α, β-unsaturated carbonyl groups to break their ether bonds and form phenolic compounds, or to convert into 3, 4-dihydrocoumarin sulfonic acid and other sulfides. Additionally, we successfully established an HPLC fingerprint method for the quantification of coumarins in ADR. The integration of SA, HCA, PCA, OPLS-DA, and other statistical methods facilitated both qualitative and quantitative analyses of coumarin quality markers in ADR. Through chemometric analysis and in vitro anti-inflammatory activity studies, byakangelicol and oxypeucedanin were identified as quality markers to distinguish between ADR and sulfur-fumigated ADR. These findings establish a scientific basis for the quality control of ADR, particularly in monitoring sulfur-fumigated samples. Although ADR has extensive applications in medicine, food, cosmetics, and health products, its underlying mechanisms remain largely unexplored. Reports indicate that sulfur fumigation significantly reduces the coumarin content, consequently diminishing the anti-inflammatory and analgesic activities of ADR. Therefore, sulfur fumigation should be avoided in the processing of ADR. There are studies that have shown that appropriate boiling could decrease the health risk of sulfur dioxide residues [1]. However, sulfur fumigation causes irreversible damage to the active ingredients; even if desulfurization technology is used or after frying, the efficacy will not be improved. Moreover, studies on the long-term stability and safety of sulfur fumigation are still lacking. Only by abandoning sulfur fumigation can the efficacy and safety of TCM really be taken into account. Therefore, seeking alternative processing methods of sulfur fumigation of ADR is the key to solving the problem. At present, the research on new processing methods of TCM, which are not easy to dry, mainly includes new drying methods, processing while fresh at the source [31]. Future research should investigate the efficacy of novel processing methods and therapeutic components and mechanisms of ADR across various applications, with the goal of discovering new active substances and expanding its potential uses.

## Figures and Tables

**Table 1:** Information on ADR collected from different areas.

No.	Place of Origin	Sulfur Fumigation	No.	Place of Origin	Sulfur Fumigation
S1	Anguo, Hebei	No	S19	Bozhou, Anhui	Yes
S2	Anguo, Hebei	No	S20	Bozhou, Anhui	Yes
S3	Anguo, Hebei	No	S21	Bozhou, Anhui	Yes
S4	Anguo, Hebei	No	S22	Hehuachi, Sichuan	No
S5	Anguo, Hebei	No	S23	Hehuachi, Sichuan	No
S6	Anguo, Hebei	No	S24	Hehuachi, Sichuan	No
S7	Anguo, Hebei	No	S25	Hehuachi, Sichuan	Yes
S8	Anguo, Hebei	No	S26	Hehuachi, Sichuan	Yes
S9	Anguo, Hebei	Yes	S27	Hehuachi, Sichuan	Yes
S10	Anguo, Hebei	Yes	S28	Hehuachi, Sichuan	Yes
S11	Bozhou, Anhui	No	S29	Hehuachi, Sichuan	Yes
S12	Bozhou, Anhui	No	S30	Hehuachi, Sichuan	Yes
S13	Bozhou, Anhui	No	S31	Hehuachi, Sichuan	Yes
S14	Bozhou, Anhui	No	S32	Yuzhou, Henan	No
S15	Bozhou, Anhui	No	S33	Yuzhou, Henan	Yes
S16	Bozhou, Anhui	No	S34	Yuzhou, Henan	Yes
S17	Bozhou, Anhui	No	S35	Yuzhou, Henan	Yes
S18	Bozhou, Anhui	No			

## Data Availability

Data are contained within the article.

## Supplementary Materials

The following supporting information can be downloaded at: https://www.mdpi.com/article/10.3390/molecules30010022/s1, Figure S1: The typical HPLC chromatograms for content determination; Figure S2: Typical average mass spectra obtained from dried ADR using MALDI MSI in CHCA-positive ion mode; Table S1: Similarity analysis of ADR fingerprint; Table S2: Contents of active ingredients in ADR (mg/g); Table S3: Identified compound from ADR using MALDI-MS/MS; Table S4: Signal strength of identified compounds in ADR before and after sulfur fumigation by MALDI-MSI; Table S5: Differential metabolites in blood of ADR before and after sulfur fumigation; Table S6: Results of linear regression, recovery rate, LOQs and LODs for the ten compounds in ADR.
