# Supplementary material for: Influence of Sulfur Fumigation on Angelicae Dahuricae Radix: Insights from Chemical Profiles, MALDI-MSI and Anti-Inflammatory Activities
Source: Molecules. 2024 Dec 25;30(1):22. doi: 10.3390/molecules30010022 (PMC11720985; doi:10.3390/molecules30010022)
Supplement: Supplementary file 1 [file molecules-30-00022-s001.zip › molecules-3331806-supplementary.pdf]

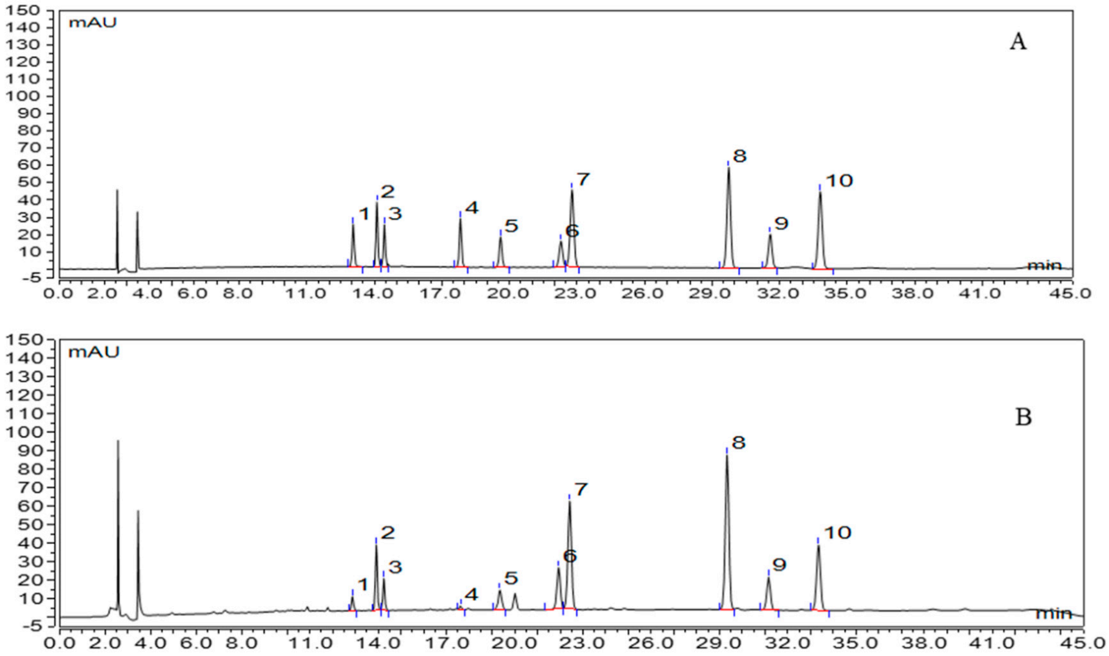

Figure S1. The typical HPLC chromatograms for content determination.

(A) Mixed standards; (B) HPLC chromatogram of ADR sample

1. xanthotoxol; 2. oxypeucedanin hydrate; 3. byakangelicin; 4. xanthotoxin; 5. bergapten; 6. byakangelicol; 7. Oxypeucedanin; 8. Imperatorin; 9. phellopterin; 10. isoimperatorin

A

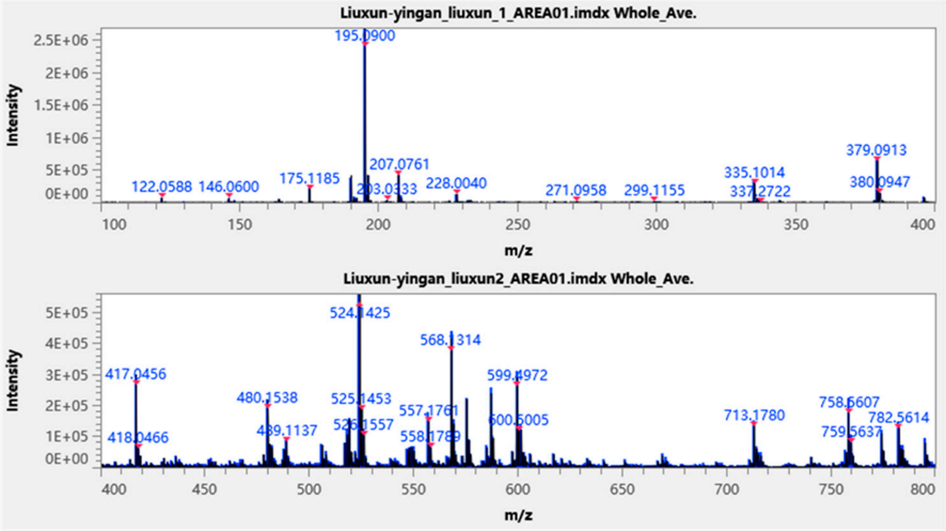

B

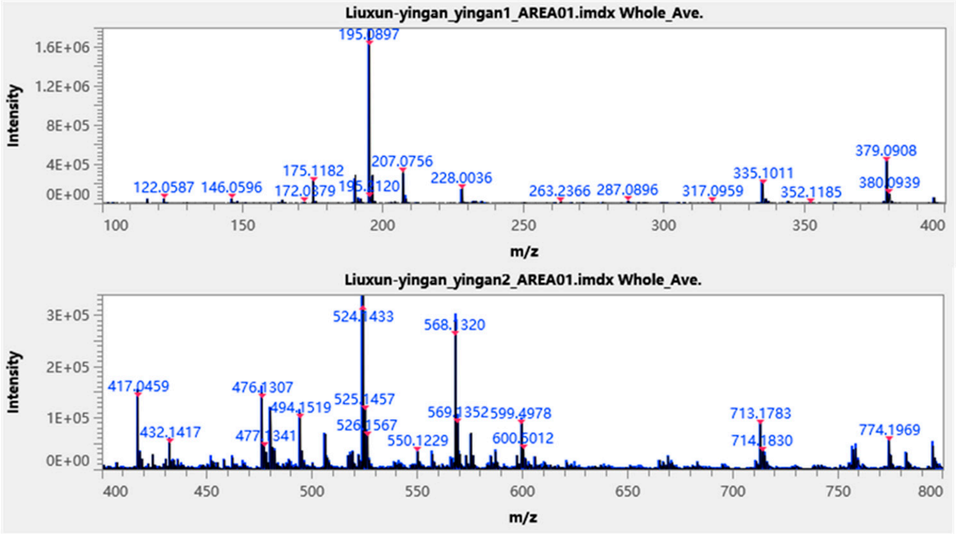

Figure S2. Typical average mass spectra obtained from dried ADR using MALDI MSI in CHCA-positive ion mode.

A: Average mass spectrum of positive ion modes for unsulfur-fumigated ADR (*m/z* 100-400; 400-800); B: Average mass spectrum of positive ion modes for sulfur-fumigated ADR (*m/z* 100-400; 400-800)

Table S1 Similarity analysis of ADR fingerprint

No.	similarity	peak number	No.	similarity	peak number	No.	similarity	peak number
S1	0.991	30	S13	0.978	31	S25	0.769	19
S2	0.994	33	S14	0.964	31	S26	0.825	19
S3	0.991	30	S15	0.955	38	S27	0.709	15
S4	0.950	31	S16	0.988	35	S28	0.793	19
S5	0.973	36	S17	0.991	32	S29	0.835	18
S6	0.995	32	S18	0.980	30	S30	0.809	19
S7	0.992	36	S19	0.802	20	S31	0.819	17
S8	0.992	31	S20	0.777	20	S32	0.991	30
S9	0.835	19	S21	0.782	20	S33	0.803	17
S10	0.837	22	S22	0.907	30	S34	0.829	18
S11	0.974	30	S23	0.971	30	S35	0.832	19
S12	0.993	31	S24	0.980	31			

Table S2 contents of active ingredients in ADR (mg/g)

No.	xanthotoxol	oxypeucedanin hydrate	byakangelicin	xanthotoxin	bergapten	byakangelicol	oxypeucedanin	imperatorin	phellopterin	isoimperatorin	total
S1	0.0124	0.226	0.096	0.0228	0.283	0.448	1.43	4.06	1.98	1.49	10.1
S2	0.0420	0.375	0.148	0.0123	0.203	0.589	1.34	3.63	1.51	1.17	9.02
S3	0.0514	0.318	0.291	0.0819	0.315	1.67	1.45	3.12	1.98	1.76	11.0
S4	0.0502	0.482	0.379	0.0228	0.282	2.26	2.06	2.76	2.04	0.96	11.3
S5	0.0445	0.449	0.216	0.0209	0.569	1.74	2.44	3.79	2.45	1.33	13.1
S6	0.0475	0.392	0.261	0.0161	0.361	1.68	1.72	3.71	1.94	1.28	11.4
S7	0.0493	0.386	0.283	0.0546	0.528	2.06	1.54	3.29	1.90	1.44	11.5
S8	0.0398	0.282	0.245	0.0179	0.274	1.72	1.33	2.83	1.38	1.19	9.30
S9	0.114	0.190	0.0757	0.0170	0.104	N.D	N.D	1.17	0.696	0.611	2.98
S10	0.121	0.118	0.213	0.0316	0.105	N.D	N.D	1.52	0.775	0.634	3.51
S11	0.0276	0.355	0.103	0.0763	0.106	0.183	0.541	2.28	1.07	0.838	5.58
S12	0.0286	0.439	0.184	0.0617	0.143	0.312	0.489	1.32	0.664	0.536	4.18
S13	0.0271	0.449	0.169	0.0186	0.142	0.372	0.515	1.98	1.14	0.99	5.81
S14	0.0543	0.463	0.196	0.073	0.189	0.369	0.653	3.23	1.15	1.10	7.48
S15	0.148	1.05	0.614	0.980	0.751	0.993	1.08	4.81	2.21	1.53	14.2
S16	0.0784	0.540	0.406	0.0193	0.221	0.833	1.53	1.77	0.618	0.916	6.93
S17	0.0417	0.631	0.204	0.0623	0.260	0.498	0.705	2.00	1.18	1.01	6.60
S18	0.0251	0.376	0.216	0.0201	0.154	0.353	0.445	1.55	0.928	0.867	4.93
S19	0.194	0.199	0.196	0.0130	0.121	N.D	N.D	0.776	0.598	0.554	2.65
S20	0.240	0.192	0.182	0.0334	0.124	N.D	N.D	0.665	0.518	0.540	2.50
S21	0.232	0.198	0.220	0.0255	0.127	N.D	N.D	0.645	0.468	0.465	2.38
S22	0.0757	0.811	0.390	0.102	0.447	0.153	0.247	2.17	1.20	0.816	6.41
S23	0.037	0.487	0.185	0.0102	0.157	0.378	0.356	1.37	0.791	0.733	4.51
S24	0.0379	0.380	0.196	0.018	0.160	0.467	0.371	1.50	0.740	0.631	4.50
S25	0.0307	0.0806	0.0551	N.D	0.0228	N.D	N.D	0.429	0.441	0.457	1.52
S26	0.0230	0.0898	0.0608	N.D	0.0304	N.D	N.D	0.869	0.701	0.505	2.28
S27	0.0212	0.0634	0.0279	N.D	0.0207	N.D	N.D	0.423	0.446	0.645	1.65
S28	0.0131	0.0915	0.0183	N.D	0.0358	0	0.0132	0.489	0.316	0.516	1.49
S29	0.0288	0.0565	0.0276	N.D	0.0232	N.D	N.D	0.706	0.378	0.328	1.55
S30	0.107	0.146	0.131	0.0134	0.0706	N.D	N.D	0.636	0.480	0.450	2.03
S31	0.0148	0.0380	0.0124	N.D	0.0071	N.D	N.D	0.433	0.384	0.233	1.12
S32	0.0478	0.488	0.285	0.0136	0.254	0.807	0.599	1.88	0.931	0.818	6.13
S33	0.0204	0.223	0.0196	N.D	0.0302	N.D	N.D	0.868	0.257	0.674	2.09
S34	0.0667	0.244	0.119	N.D	0.125	N.D	N.D	1.67	1.11	1.04	4.38
S35	0.0180	0.258	0.0378	N.D	0.153	N.D	N.D	1.44	0.557	0.790	3.25

Table S3 Identified compound from ADR using MALDI-MS/MS

No.	compound	formula	adductions	theoretical	measured	error, ppm
1	xanthotoxol	$C_{11}H_6O_4$	$[M+H]^+$	203.034	203.033	-4.9
2	xanthotoxin	$C_{12}H_8O_4$	$[M+H]^+$	217.050	217.048	-9.2
3	5-methoxy-8-hydroxy-psoralen	$C_{12}H_8O_5$	$[M+H]^+$	233.044	233.043	-4.3
4	osthole	$C_{15}H_{16}O_3$	$[M+H]^+$	245.117	245.116	-4.1
5	imperatorin/isoimperatorin/ alloimperatorin/alloisoimperatorin	$C_{16}H_{14}O_4$	$[M+H]^+$	271.096	271.096	0
			$[M+K]^+$	309.053	309.051	-6.5
6	(R)-Peucedanol	$C_{15}H_{18}O_5$	M+Na	301.105	301.111	19.9
7	Phellopterin	$C_{17}H_{16}O_5$	M+K	339.063	339.062	-2.9
8	Oxypeucedanin hydrate	$C_{16}H_{16}O_6$	M+H	305.102	305.104	6.6
9	Oxypeucedanin/Pangelin	$C_{16}H_{14}O_5$	M+H	287.089	287.091	7.0
10	Tert-O-Methylheraclenol	$C_{17}H_{18}O_6$	M+H	319.118	319.111	-21.9
11	Byakangelicol	$C_{17}H_{16}O_6$	M+K	355.058	355.056	-5.6
12	Scoparone	$C_{11}H_{10}O_4$	M+H	207.065	207.076	53.1
13	L-proline	$C_5H_9NO_2$	M+H	116.071	116.069	-17.2
14	L-arginine	$C_6H_{14}N_4O_2$	M+H	175.119	175.118	-5.7
15	Phosphorylcholine	$C_5H_{14}NO_4P$	M+H	184.073	184.073	0.0
16	kynurenate	$C_{10}H_7NO_3$	M+K	228.006	228.004	-8.8
17	phenylacetylglycine	$C_{10}H_{11}NO_3$	M+K	232.038	232.036	-8.6
18	glycerophosphorylcholine (GPC)	$C_8H_{20}NO_6P$	M+H	258.110	258.110	0
19	Melatonin	$C_{13}H_{16}N_2O_2$	M+K	271.085	271.096	40.6
20	isoquercitrin	$C_{21}H_{20}O_{12}$	M+Na	487.085	487.104	39.0
21	Naringin	$C_{27}H_{32}O_{14}$	M+Na	603.168	603.185	28.2
22	biliverdin	$C_{33}H_{34}N_4O_6$	M+K	621.212	621.188	-38.6
23	Hesperidin	$C_{28}H_{34}O_{15}$	M+Na	633.179	633.187	12.6

Table S4 Signal strength of identified compounds in ADR before and after sulfur fumigation by MALDI-MSI

No.	Identified compound	sulfur	no sulfur
1	Byakangelicol	17.604	197.474
2	L-proline	27.426	1195.648
3	Xanthotoxin	29.767	100.068
4	Oxypeucedanin hydrate	58.609	226.104
5	Phellopterin	100.478	175.916
6	Oxypeucedanin/Pangelin	188.092	1038.996
7	Tert-O-Methylheraclenol	221.285	63.934
8	Osthole	234.002	83.405
9	Phosphorylcholine	238.741	184.334
10	glycerophosphorylcholine (GPC)	302.101	229.747
11	(R)-Peucedanol	326.842	305.481
12	Melatonin	389.383	263.253
13	Xanthotoxol	766.178	554.057
14	5-Methoxy-8-hydroxy-psoralen	892.335	778.024
15	phenylacetylglycine	987.097	913.94
16	kynurenate	2606.653	4080.183
17	L-arginine	4327.004	5288.249

Table S5 Differential metabolites in blood of ADR before and after sulfur fumigation

NO.	Compounds	classes	Formula	Mean peak area of ADR before sulfur fumigation	Mean peak area of ADR after sulfur fumigation
1	2,5-Dihydroxybenzoic acid	Phenolic acids	$C_7H_6O_4$	23267	135277
2	3-hydroxyphenylacetic acid	Phenolic acids	$C_8H_8O_3$	15140	1156
3	4-Hydroxyphenylacetic acid	Phenolic acids	$C_8H_8O_3$	7588	441
4	Benzoic acid	Phenolic acids	$C_7H_6O_2$	17283	120475
5	Diisooctyl Phthalate	Phenolic acids	$C_{24}H_{38}O_4$	23626	11195
6	Homovanillic acid sulfate	Phenolic acids	$C_9H_{10}O_7S$	5269	29226
7	Methyl 4-hydroxybenzoate	Phenolic acids	$C_8H_8O_3$	93978	8842
8	Phenyl acetate	Phenolic acids	$C_8H_8O_2$	4487	86224
9	p-Coumaric acid	Phenolic acids	$C_9H_8O_3$	25996	2821
10	4,4'-dihydroxy-2,6-dimethoxydihydrochalcone	Flavonoids	$C_{17}H_{18}O_5$	3231	352
11	Xanthoangelol D	Flavonoids	$C_{21}H_{22}O_5$	935	14455
12	4-Hydroxycoumarin	Coumarins	$C_9H_6O_3$	23930	4405
13	6,7-Dihydroxy-4-methylcoumarin	Coumarins	$C_{10}H_8O_4$	17726	1606
14	Heraclenol	Coumarins	$C_{16}H_{16}O_6$	1667155	492211
15	Oxypeucedanin	Coumarins	$C_{16}H_{14}O_5$	138363	2710
16	Oxypeucedanin hydrate	Coumarins	$C_{16}H_{16}O_6$	137968	42784
17	Rutaretin	Coumarins	$C_{14}H_{14}O_5$	213583	20521
18	1-Indanone	Ketones compounds	C_9H_8O	4548	26004
19	2,5-Dihydroxybenzaldehyde	Aldehyde compounds	$C_7H_6O_3$	120637	21618
20	3-Methylbenzaldehyde	Aldehyde compounds	C_8H_8O	5784	526
21	Vanillin; 4-Hydroxy-3-Methoxybenzaldehyde	Aldehyde compounds	$C_8H_8O_3$	449542	142223
22	2-Amino-4,5-dihydro-1H-imidazole-4-acetic acid	Alkaloids	$C_5H_9N_3O_2$	13244	949873
23	6-Methylnicotinamide	Alkaloids	$C_7H_8N_2O$	46163	8184
24	Echinopsine	Alkaloids	$C_{10}H_9NO$	177171	384626
25	Indole-3-lactic acid	Alkaloids	$C_{11}H_{11}NO$	57391	24541
26	L-Carnitine	Alkaloids	$C_7H_{15}NO_3$	61917	6893
27	16,23:16,30-Diepoxydammar-24-ene-3,20-diol	Triterpene	$C_{30}H_{48}O_4$	28283	3586
28	2,3-Dihydroxyurs-12-en-29-oic acid	Triterpene	$C_{30}H_{48}O_4$	31320	5112
29	3,19-Dihydroxyurs-12-en-28-oic acid	Triterpene	$C_{30}H_{48}O_4$	37721	5285
30	Virgatic acid	Triterpene	$C_{30}H_{46}O_4$	36044	5318

Table S6 Results of linear regression, recovery rate, LOQs and LODs for the ten compounds in ADR

compounds	equation	r	linear range/ng	recovery, %	recovery RSD, %	LOQ (ng)	LOD (ng)
xanthotoxol	y = 122.76 x - 0.05	1.00	2.40 ~ 242	101	1.50	0.485	0.146
oxypeucedanin hydrate	y =78.76 x - 0.03	1.00	4.70 ~ 472	98.3	1.90	0.944	0.283
byakangelicin	y = 51.16 x - 0.03	1.00	4.70 ~ 474	102	0.90	0.948	0.284
xanthotoxin	y = 133.62 x + 0.00	1.00	2.40 ~ 243	101	1.30	0.486	0.146
bergapten	y = 106.19 x - 0.02	1.00	2.40 ~ 239	99.3	1.00	0.478	0.144
byakangelicol	y = 57.62 x - 0.02	1.00	4.60 ~ 463	100	0.90	0.926	0.278
oxypeucedanin	y = 85.81 x + 0.00	1.00	10.0 ~ 998	98.3	1.30	2.00	0.599
imperatorin	y = 106.76 x + 0.08	1.00	10.3 ~ 1032	99.9	1.00	2.06	0.619
phellopterin	y = 64.33 x + 0.02	1.00	5.80 ~ 578	101	0.50	1.16	0.347
isoimperatorin	y = 93.56 x + 0.28	1.00	10.0 ~ 998	99.8	0.90	2.00	0.598
